# Phage diversity in One Health

**DOI:** 10.1042/EBC20240012

**Published:** 2024-12-17

**Authors:** Hannah V. Pye, Revathy Krishnamurthi, Ryan Cook, Evelien M. Adriaenssens

**Affiliations:** 1 Quadram Institute Bioscience, Norwich Research Park, Norwich, NR4 7UQ, UK; 2 Centre for Microbial Interactions, Norwich Research Park, Norwich, NR4 7UG, UK

**Keywords:** Antimicrobial Resistance, Bacteriophages, Diversity, One Health, Viruses

## Abstract

One Health aims to bring together human, animal, and environmental research to achieve optimal health for all. Bacteriophages (phages) are viruses that kill bacteria and their utilisation as biocontrol agents in the environment and as therapeutics for animal and human medicine will aid in the achievement of One Health objectives. Here, we assess the diversity of phages used in One Health in the last 5 years and place them in the context of global phage diversity. Our review shows that 98% of phages applied in One Health belong to the class *Caudoviricetes*, compared to 85% of sequenced phages belonging to this class. Only three RNA phages from the realm *Riboviria* have been used in environmental biocontrol and human therapy to date. This emphasises the lack in diversity of phages used commercially and for phage therapy, which may be due to biases in the methods used to both isolate phages and select them for applications. The future of phages as biocontrol agents and therapeutics will depend on the ability to isolate genetically novel dsDNA phages, as well as in improving efforts to isolate ssDNA and RNA phages, as their potential is currently undervalued. Phages have the potential to reduce the burden of antimicrobial resistance, however, we are underutilising the vast diversity of phages present in nature. More research into phage genomics and alternative culture methods is required to fully understand the complex relationships between phages, their hosts, and other organisms in the environment to achieve optimal health for all.

## Introduction

The advent of ‘One Health’ occurred in early 2003 during the emergence of severe acute respiratory disease (SARS) and was one of the first examples of how both human and animal related diseases can have catastrophic effects on food supplies and the economy [1]. Although there are many definitions of One Health, it is generally regarded as the multidisciplinary and collaborative approach to achieving optimal health for all by recognising the interconnected relationship between humans, animals, and the environment. The antimicrobial resistance (AMR) crisis has exacerbated the need for multidisciplinary collaboration to identify viable alternative therapies and has resulted in a renewed interest in using bacteriophages (phages) in addition to antimicrobials to treat and prevent bacterial infections.

## Global bacteriophage diversity

Virulent phages are viruses that specifically bind to and kill bacteria. They are naturally occurring in the environment and have a high amount of morphological and genetic diversity. At the time of writing, phages are taxonomically classified into four out of the six established realms of viruses, belonging to seven kingdoms and eight phyla [2–4]. Recent virome studies (including metatranscriptomics and metagenomics) have shown massive expansions of the isolated virosphere, with the current IMG/VR database tallying more than 5 million high-confidence virus genome predictions, grouped into 2.9M viral operational taxonomic units (vOTUS) [5].

**Figure 1:**
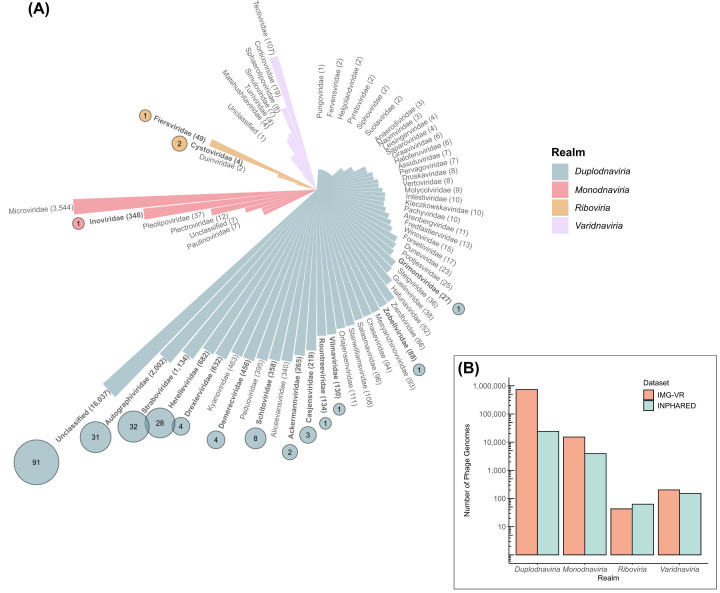
Phage genomes in sequence databases and One Health applications (**A**) Bars represent the number of phage genomes in the INPHARED database (as of September 2024), grouped by family and coloured by realm in descending order. The number of genomes per family is noted in parentheses. Taxa without known bacterial hosts (e.g., *Adnaviria*) were excluded. Therapeutic phage families with available genomes are marked with coloured circles, scaled and labelled by genome count. (**B**) Bacteriophage genome counts in IMG/VR (v4.1) and INPHARED databases, grouped by realm. IMG/VR sequences were filtered to include only those with bacterial hosts and therefore exclude those with unknown hosts that are predicted to be phages. Data are provided in Supplementary Table S1.

The majority of isolated phages (Figure 1A) belong to the realm *Duplodnaviria*, which are tailed dsDNA phages containing a HK97-like major capsid protein. All dsDNA phages can be classified into the class *Caudoviricetes* within the *Uroviricota* phylum [6]. Within this class, 52 families of bacteria-infecting viruses have been described to date, comprising 4,863 species. Phages belonging to the *Monodnaviria* realm are ssDNA viruses with either filamentous (*Inoviridae*, *Plectroviridae*, *Paulinoviridae* families in the order *Tubulavirales*) or icosahedral (family *Microviridae* in the order *Petitvirales*) morphologies. Phages in the realm *Varidnaviria* consists of dsDNA viruses in the families *Tectiviridae*, *Corticoviridae, Autolykiviridae* and *Matsushitaviridae* (with the exception of the single-stranded FLiP viruses in the family *Finnlakeviridae*) that are characterised by homologous major capsid proteins with one or two jelly fold β-barrel domains [6,7]. Phages classified into the realm *Riboviria* are either dsRNA viruses (*Cystoviridae*) or positive-sense ssRNA viruses unified into the class *Leviviricetes* and six families [8,9]. These numbers pale in comparison with the number of vOTUS and family-rank clades that have been reported in the human gut alone where more than 168k species-level clusters are available from the Unified Human Gut Virus Catalog (comprising 12 high-throughput studies of the human gut such as the Gut Phage Database, the Metagenomic Gut Virus Compendium, the Human Virome database and the Atlas of Infant Gut DNA Virus Diversity) (https://github.com/snayfach/UHGV) [5,10–20]. Similarly, thousands of novel species-level vOTUs spread across realms have been described in various environments, such as the marine environment [21–24], soils [25–27], and sewage [28,29], expanding the diversity of specifically *Caudoviricetes* and *Leviviricetes* phages exponentially.

Phage genomes can vary vastly in size, further highlighting their diversity. For example, the smallest isolated phage with a complete genome is *Lactobacillus* phage ADMH1Phi, which is 1,761 bp in size, compared to the largest cultured phage genome available in the INPHARED database (curated database of complete genomes of isolated phages), which is *Bacillus* phage G at 497,513 bp [30–32]. Some large phages with genomes >540,000 bp have been identified in metagenomic datasets from the human gut and marine environments and have been designated “Megaphages”, however these phages remain uncultivated at present [21,33,34].

As of September 2024, 28,468 complete phage genomes have been sequenced and are incorporated into the INPHARED database, after filtering out taxa for which no bacterial host is known [35]. Approximately 85% of these genomes are dsDNA phages belonging to the realm *Duplodnaviria* (Figure 1) [35]. The second highest number of genomes belong to the *Monodnaviria* realm (∼14%), with very few genomes belonging to the other realms.

In this review, we summarise the diversity of a selection of phages that have been applied for use in humans, animals and the environment mostly occurring within the last 5 years. A full meta-analysis of phages used for advancing One Health is beyond the scope of this review, however we have included a representative selection of sequenced phages used in each sector where possible.

## Phages for food and environmental use

The overuse and misuse of antibiotics in agriculture and livestock management has led to an increase in antibiotic resistant bacteria (ARB) and antibiotic resistance gene (ARG) accumulation in the environment [36,37]. Phages can be applied to crops to kill pathogenic bacteria and can also be used to disinfect food and the food production environment during processing. Interest in using phages as a biocontrol for foodborne pathogens is expanding due to their ability to kill contaminating bacteria without affecting the taste, colour, smell, or texture of the product, unlike some more conventional food preservation methods [38–40]. A compilation of phage products commercially available for application in the food industry can be found on the phage products database (https://www.bacteriophage.news/phage-database).

Several phage products have been approved by the US Food and Drug Administration (FDA) for food application in the United States and Europe, such as ListShield™ (Intralytix) and Phageguard L (formerly Listex™) for reducing the presence of *Listeria monocytogenes* on various ready-to-eat products and SalmoFresh™ (Intralytix) for preventing *Salmonella* contamination of food products [39,41,42]. ListShield™ comprises a cocktail of six phages which all belong to the family *Herelleviridae* and genus *Pecentumvirus* (LIST-36, LMSP25, LMTA-34, LMTA-57, LMTA-94 and LMTA-148) [39,41]. Phageguard L contains only a single broad-host range phage (P100), which also belongs to the *Pecentumvirus* genus and *Herelleviridae* family [43]. Notably, all phage products currently on the market which aim to reduce *L. monocytogenes* in food products belong to this same genus and family.

SalmoFresh™ is a cocktail of six phages (SPT-1, STML-198, SSE-121, SBA-1781, SKML-39 and STML-13-1) that belong to different families and genera [44]. Two of the phages included in the cocktail (SPT-1 and SBA-1781) belong to the *Felixounavirus* genus, however the family is currently unclassified in the class *Caudoviricetes*. Two of the phages (SKML-39 and STML-13-1) belong to different genera, *Agtrevirus* and *Kuttervirus* respectively, within the *Ackermannviridae* family. The fifth phage (STML-198) belongs to the *Straboviridae* family and *Gelderlandvirus* genus. The final phage in the cocktail (SSE-121) has been classified as belonging to the *Seunavirus* genus, but the family remains unclassified. SalmoFresh™ has been shown to reduce *Salmonella* contamination on lettuce and sprouts by up to threefold and was shown to be more effective on produce at refrigerated temperatures (2–10°C) that at 25°C [45]. Similarly, SalmoFresh™ has been successfully applied to reduce *Salmonella enterica* burden in whole and fresh-cut cucumbers [46]. A very similar *Salmonella* phage product called SalmoLyse™ (Intralytix) consists of four phages common to SalmoFresh™ (SPT-1, STML-198, SSE-121 and SBA-1781) and two unique phages (SEML-239-1 and SNN-387) [44]. The genomes of *Salmonella* phage SEML-239-1 and SNN-387 do not appear to be publicly available, however electron micrographs of these phages indicate that they are both tailed phages with myovirus-like morphology [44].

Commercial products targeting Shiga-Toxin producing *Escherichia coli* (STEC) O157:H7 have also been approved by the FDA, including EcoShield™ (Intralytix) and EcoShield™ PX (Intralytix), which both contain three to eight obligately lytic phages. EcoShield PX is the most recent STEC targeting phage product from Intralytix and it contains three phages (ECML-117, ECML-359 and ECML-363) which belong to the genera *Wifcevirus, Gaprivervirus* and *Tequatrovirus*, respectively, and are nonenveloped dsDNA phages with contractile tails [47]. Additional products used for the control of *E. coli* in the food industry include Phageguard E and Phageguard E Hides, the latter of which is designed to be applied to animal hides (preharvest) to control *E. coli* O157 (https://phageguard.com/solutions/e-coli). Recently, a patent has been granted in China for a phage product to treat *Aeromonas hydrophila*, which is an emerging foodborne pathogen commonly associated with seafood and causes human gastroenteritis [48]. This is the first example of a jumbo phage (> 200 kbp) being used commercially to treat a foodborne pathogen and belongs to a novel genus *Chaoshanvirus* [48].

Phage products implemented for the biocontrol of plant pathogens include Biolyse™ (APS Biocontrol Ltd.) targeting *Pectobacterium* and *Dickeya* spp. to prevent soft rot in tubers, however the phage composition is not disclosed [49]. Erwiphage™ (Erwiphage PLUS) is a product for the biocontrol of fire blight disease in *Rosacea* plants and encompasses two dsDNA jumbo phages (PhiEaH1 and PhiEaH2), which belong to the *Iapetusvirus* and *Erskinevirus* genera within the class *Caudoviricetes* [50,51].

AgriPhage (Omnilytics) is an example of a commercial phage product consisting of a cocktail of six phages approved by the FDA for the biocontrol of *Xanthomonas campestris* pv.* vesicatoria* and *Pseudomonas syringae* pv. *tomato*, which are the causative agents of bacterial spot disease of peppers and tomatoes [52]. Bacteriophage phi6 (Φ6) is an example of a dsRNA phage of the *Cystoviridae* family within the realm *Riboviria*, which has been used commercially in agriculture [53,54]. Since its discovery in 1973, phi6 has been used for the biocontrol of halo blight in legumes and bacterial canker in kiwifruit, caused by *P. syringae* pathovars *phaseolicola* and *actinidiae*, respectively [53–55]. Most recently phage phi6 has served as a surrogate for SARS-CoV-2 and other animal viruses, due to their structural similarity [56–58].

## Phages for human use

Followed by a few successful human case reports of phage therapy in 1919 and establishment of the George Eliava Institute of Bacteriophages, Microbiology and Virology (Eliava Institute) in 1923, Felix d'Herelle reported a preliminary account of the successful use of phage therapy in a cholera cohort in India during 1927 [59–61]. The emergence of antibiotics and the knowledge gap on the nature of phages stalled the therapeutic use of phages in some countries during the 1930’s. However, in Eastern European countries like Georgia and Poland, phages are considered a pharmaceutical for treating bacterial infections and are routinely used [62].

To date, phage therapy is considered as a viable treatment option in compassionate human cases, owing to the rise of multidrug resistant bacterial infections and decline in the antibiotic discovery pipeline. A recent retrospective observational analysis of 100 cases of personalised phage therapy for 14 difficult-to-treat infections revealed that in approximately 77% of cases clinical improvement was achieved and in ∼61% of cases complete eradication of the bacterium occurred with eradication more probable when concurrent antibiotics were given [63] (Supplementary Table S2). There are no phage therapy products on the market that have received approval by the FDA, however, phages have been granted emergency status for use in instances where antibiotic treatment has failed. Currently, there are more than 40 ongoing clinical trials registered at clinicaltrials.gov at various stages for validating the safety and efficacy of phage cocktail products in treating human infections (Supplementary Table S3). Phages used in active trials are kept trade secret, for instance; AP-SA02 (Armata), VRELysin® (Intralytix), DUOFAG® (MB Pharma) etc, for which the composition of phages were not available (Supplementary Table S3).

In this section, we describe the diversity of a selection of phages that are currently in use for treating bacterial human infections. At present, phage therapy is used on a compassionate basis for treating infections caused by multidrug resistant pathogens in conditions like surgical wounds, burn wounds, chronic lung infections, urinary tract infections, irritable bowel syndrome, chronic rhinosinusitis and acne (Supplementary Table S2 and S3).

*E. coli* is described as one of the difficult-to-treat pathogens in infections caused post-surgery and in recurrent prostate or urinary tract infections [63–65]. Phages used to treat human *E. coli* infections are very diverse within the class *Caudoviricetes* (Figure 1) belonging to the families or subfamilies *Autographiviridae* (*Vectrevirus, Berlinvirus, Kayfunavirus*), *Straboviridae* (*Tequatrovirus, Krischvirus*), *Schitoviridae* (*Gamaleyavirus*), *Drexlerviridae* (*Rogunavirus*), *Demerecviridae* (*Tequintavirus*), unclassified* Caudoviricetes, Gordonclarkvirinae* (*Kuravirus, Suseptimavirus*), *Stephanstirmvirinae* (*Justusliebigvirus*), *Vequintavirinae* (*Vequintavirus*) and *Ounavirinae* (*Felixounavirus*). Similarly, phages of *Shigella* spp. are from families *Straboviridae* (*Tequatrovirus, Mosigvirus*), *Demerecviridae* (*Tequintavirus*) and *Drexlerviridae* (*Tunavirus*) [63–69]. Due to the close relatedness of *E. coli* and *Shigella* bacteria, there is an overlap in the diversity of their phages, specifically in the families *Straboviridae* and *Drexlerviridae*. However, the phages that are part of the ShigActive™ cocktail (SHSML-52-1, SHFML-11, SHSML-45, SHFML-26, SHBML-50-1) belong to genera (*Tunavirus*, *Mosigvirus*) only used therapeutically for shigellosis, whereas the *E. coli* phages in these genera are not used therapeutically at this time.

*Staphylococcus aureus* is a major cause of infections in prosthetic joints, diabetic foot ulcers, skin burns, chronic lung infections and infections post organ transplantation [63–65,70–81]. More than 20 phages are currently in use to treat multidrug resistant *Staphylococcus* spp., including the Eliava Institute’s phage ISP. Phage ISP belongs to the *Herelleviridae* family, which is the family that encompasses most known *Staphylococcus* phages [82]. Recently in the UK, ten patients with diabetic foot infections at risk of foot amputation were treated with a topical formulation of phage ISP. Nine of the patients treated with ISP exhibited clinical improvement. This case study represents the largest application of phage for treating human infections in the UK to date [70]. *Staphylococcus* phages broadly come from the families of either *Herelleviridae* (*Kayvirus, Sepunavirus*), *Autographiviridae* (*Kayfunavirus, Pokrovskaiavirus*), *Rountreeviridae* (*Rosenblumvirus*) or genera that are unclassified within the class *Caudoviricetes* [63–65,68–70,75,77–81,83–85].

Approximately 15 *Enterococcus* phages are in use including the multiphage cocktails from the Eliava Institute, named Pyophage and Intestiphage, to treat infections following periprosthetic surgery, transplantations, and musculoskeletal infections caused by *Enterococcus* spp. [63–65,86–88]. Phages of *Enterococcus* spp. belong to the *Herelleviridae* family (*Schiekvirus, Kochikohdavirus*), or unclassified *Caudoviricetes* (*Saphexavirus*, unclassified genus) [63–65,68,86–88].

*Klebsiella* colonisation in the lungs and postsurgical sites are often difficult to treat with antibiotics and sometimes even with phage therapy due to the heterogeneity of the pathogen [64,89–92]. Some of the phages used in treating *K. pneumoniae* includes the families *Autographiviridae* (*Drulisvirus, Przondovirus*, unclassified *Melnykvirinae*, unclassified *Autographiviridae*), *Drexlerviridae* (*Webervirus*), *Straboviridae* (*Jiaodavirus, Slopekvirus*) or unclassified *Caudoviricetes* (*Jedunavirus*, unclassified genera) [64,65,68,83,91–97].

*Pseudomonas aeruginosa* is one of the pathogens listed on WHO's multidrug resistant pathogen list and is often identified in recalcitrant infections resistant to existing antibiotics currently in use [98]. The majority of lung infections and other difficult-to-treat infections in hospital settings are due to the *Pseudomonas* burden in the site of infection, as reviewed previously [99]. The recent retrospective case analysis compiled by the Belgium researchers, reveals that 22 patients were treated with *P. aeruginosa* phage 14-1 in a monophage preparation, and an additional 14 patients were treated with a phage cocktail called BFC-1, which incorporated phages 14-1, PNM and ISP. *P. aeruginosa* phage 14-1 is taxonomically classified into the *Pbunavirus* genus, an unclassified genus in the class *Caudoviricetes*. There were two other phages used to treat patients with *Pseudomonas* infections in this analysis, which also belonged to the *Pbunavirus* genus (DP1 and Phage C). All these *Pbunaviruses* had genome sizes of ∼66,000 bp and a myovirus morphotype [63]. Bacteriophages so far used in treating human *Pseudomonas* infections have been isolated from the families *Schitoviridae* (*Litunavirus*), *Zobellviridae* (*Paundecimvirus*), *Autographiviridae* (*Phikmvvirus*), *Fiersviridae* (*Perrunavirus*), *Cystoviridae* (*Cystovirus*), and unclassified *Caudoviricetes* (*Nipunavirus, Pbunavirus, Pakpunavirus, Nankokuvirus, Phikzvirus, Bruynoghevirus*, unclassified genera) [63–65,68,69,83,88,100–118].

The only phages used in treating non-tuberculous *Mycobacteria* were originally temperate phages made lytic by precise deletion of repressor genes or by isolation of host range mutants that effectively lyse the host [119,120]. At least five phages have been reported in treating *Mycobacterium abscessus* and are isolated from unclassified families (*Mapvirus, Fionnbharthvirus, Liefievirus, Timquatrovirus*) and the *Vilmaviridae* family (*Faithunavirus*) in the class *Caudoviricetes* [119,120].

Few other phages that are commonly used in phage therapy to treat prosthetic joint infections are all from the class *Caudoviricetes. Enterobacter* phages were from the family *Straboviridae* (*Krischvirus, Pseudotevenvirus*) [65], *Acinetobacter* phages from the families *Autographiviridae* (*Friunavirus, Daemvirus*) and the unclassified genus *Saclayvirus* [92,121]. Phages against *Achromobacter* were from *Schitoviridae* (*Jwalphavirus*), and unclassified *Caudoviricetes* (*Steinhofvirus* and unclassified genus) [122]. The only known phage to treat *Proteus mirabilis* infection is phage pPM_01 included in the Pyophage cocktail belonging to an unassigned genus *Lavrentievavirus* [63]. Another notable phage used to treat a patient with multiple complications including *Stenotrophomonas maltophilia* infection, is phage vB_SmeS_BUCT700 of the family *Autographiviridae* [63].

Phages belonging to the realms *Riboviria* and *Varidnaviria* make up ∼1% of the total number of complete phage genomes in the INPHARED database. However, interest in using ssDNA and RNA phages as therapeutics is increasing due to the advancement of metagenomics and metatranscriptomics, which has unveiled their potential use as medical tools [123]. PhiYY is the only dsRNA phage that is currently tested in humans to treat *Pseudomonas* infections, belonging to the *Cystovirus* genus and *Cystoviridae* family at the time of writing this review [104]. The inclusion of ssDNA and RNA phages that have been applied in biotechnological applications related to human medicine is out of the scope of this review, as these are mainly genetically engineered phages. However, Nguyen and colleagues (2023) have highlighted the major contributions of RNA and ssDNA phages in the advancement of molecular and synthetic biology, wherein these phages have predominantly been used for gene editing, bacterial detection and control, vaccine development and cancer treatment, amongst other applications [124–126].

## Phages for animal use

An EU-wide ban on the prophylactic use of antimicrobials in animal husbandry came into force in 2022, which has initiated a resurgence in interest in the use of phages for veterinary medicine [127,128]. Nine veterinary phage products have been approved by the FDA targeting the most common pathogens infecting livestock and companion animals, including *Salmonella, E. coli* and *S. aureus* [51]. One phage product called INT-401™ (Intralytix) combines five phages to control necrotic enteritis in broiler chickens, which is caused by the bacterium *Clostridium perfringens*. In the study, administering the INT-401 phage cocktail via drinking water/feed or oral gavage reduced the mortality of broiler chickens spiked with *C. perfringens* by 92% [129]. Of the five phages used in the cocktail (CPAS-7, CPAS-12, CPAS-15, CPAS-16, and CPLV-42), only the genome of CPAS-15 has been deposited into the NCBI database and belongs to a currently unclassified order, family, and genus of the class *Caudoviricetes* (Figure 1A). A further 29 non-FDA approved animal phage products have been commercialised and they are well-documented in the recent review by Huang *et* *al*., 2022.

Aquaculture is heavily impacted by bacterial diseases and leads to high mortality rates and significant economic losses in commercially farmed fish. Phages have been explored as a viable alternative to using antibiotics to treat fish-related diseases, including vibriosis, aeromonasis, mycobacteriosis and ulcer disease. The application of phages in aquaculture is advantageous as they specifically target their host bacterium, can self-replicate in the presence of their host and are easy to administer in fisheries. Numerous products have been commercialised for use in aquaculture and are detailed in the phage product database. CUSTUS®YRS (ACD Pharma) can be added to water to protect against *Yersinia ruckeri* (https://stim.uk/r-d/bacteriophages). BF-VP (APB Bio) and LUXON (Phagelux) (https://www.bacteriophage.news/database/phagelux) are both aqueous solutions that can be added to water to protect against *Vibrio parahaemolyticus*, especially in shrimp farms where there is a high economic burden caused by Early Mortality Syndrome (EMS) [130]. Unfortunately, the phage composition of the aforementioned products is not publicly available. Proteon pharmaceuticals have developed a phage cocktail, called BAFADOR, which targets *Pseudomonas* and *Aeromonas* infections in the European Eel. BAFADOR is a phage cocktail consisting of seven phages, three of which target *Aeromonas hydrophila* (50AhydR13PP, 60AhydR15PP, 25AhydR2PP) and four which infect *Pseudomonas fluorescens* (22PfluR64PP, 67PfluR64PP, 71PfluR64PP, 98PfluR60PP) [131].

Despite the interest in phage therapy for human and animal use, there is currently a lack of literature detailing phage usage in companion animals. One of the earliest reports of phage therapy being used in companion animals occurred in 2006 when a dog was treated for chronic otitis caused by *P. aeruginosa*, however details on the phage preparation used to treat the animal were not disclosed in the publication [132]. Following on from this success, a phage therapy clinical trial was initiated that included 10 dogs affected with *P. aeruginosa* associated chronic otitis. The dogs were given a topical treatment comprised of 6 phages (BC_BP_01 to BC_BP_06), and after treatment all dogs exhibited clinical improvement [133]. Unfortunately, the genome sequences of the phages used in the trial are not publicly available.

A case report of a successful phage-antibiotic veterinary therapeutic was recently described, wherein a cat with an implant-associated *P. aeruginosa* infection was treated topically with phage PASB7 in conjunction with intravenous administration of ceftazidime over seven weeks. The phage-antibiotic treatment resulted in complete closure of the wound and eradication of the infecting pathogen [134]. Genomic sequencing of PASB7 phage classified it into the *Schitoviridae* family within the *Caudoviricetes* class and TEM imaging showed the phage encompasses a short tail (Figure 1A). Another case-report detailing the successful treatment of a *P. aeruginosa* wound infection in a dog was also recently described [135]. In this case, the dog was treated with a commercial phage cocktail, called Picobacteriophage polyvalent polyphage—Sixtaphage (MIKROGEN), which incorporates six phages targeting *Staphylococcus* spp., *Streptococcus* spp., *Proteus* spp., *P*. *aeruginosa* and enteropathogenic *E. coli* (EPEC), although the exact composition of the phage cocktail remains proprietary knowledge [135].

## Discussion

The most recent International Committee on Taxonomy of Viruses (ICTV) update implemented the proposal that phages should be classified according to their genome rather than the historical morphology-based method [136]. However, when conducting our literature search on the application of phages over the past 5 years it became clear that many of the phages being applied for use in the veterinary, environmental, or human medicine sectors had no genome sequencing data available, primarily due to nondisclosure from the producers. Whole genome sequencing data is paramount to understanding the fundamental biology of the phage, the presence of any deleterious or unwanted genes, and gives an indication of phage lifestyle, which is important when applying the phage for biocontrol of pathogens or for eradicating human infections. Analysing the genomes of lytic phage can also identify whether the phage has any transduction ability and can identify genes of concern, such as toxins or antimicrobial resistance genes which could be mobilised to other bacteria, contributing to the spread of antimicrobial resistance.

From our literature review, it is apparent that phage products intended on being used for One Health are dominated by dsDNA phages from the class *Caudoviricetes* (Figure 1) making up 98% of phages reviewed here. The disproportionately high number of phages belonging to *Duplodnaviria* is indicative of isolation bias, as traditional phage culture methods favour the isolation of dsDNA phages. One of the most common methods of isolating phages is from wastewater, however this is often restricted to phages of gastrointestinal pathogens and environmental bacteria, thus efforts to isolate virulent phages for low abundant bacteria in wastewater are often unsuccessful. *Staphylococcus epidermidis* is an example of a bacterium with very few therapeutic phages available. Efforts to isolate *S. epidermidis* from wastewater and hospital sewage remains challenging, however recent success has been achieved by using skin microbiome samples as opposed to wastewater, indicating the need for targeted phage isolation methodologies rather than a ‘one size fits all’ approach [137,138].

When assessing the overall diversity of the phages used in One Health applications, phages of certain families are encountered more than others. For example, phages of the family *Autographiviridae* are most commonly used, followed by the families *Straboviridae* and *Herelleviridae*. Their increased representation could be attributed to the diversity of their hosts, with the latter family containing phages infecting Gram-positive bacteria that are potential pathogens *(Listeria* and *Staphylococcus*), and the former two families comprising phages infecting Enterobacterales bacteria, which are the most common targets for phage therapy. The skew could also be caused by selection bias, where taxonomically related phages have certain shared characteristics that make them ideal candidates for phage-based applications such as broad host range, rapid killing *in vitro* and large burst size.

The phages isolated as biocontrol agents against *L. monocytogenes* that were summarised in this review showed lack of genomic diversity as they were all taxonomically classified into the *Herelleviridae* family and *Pecentumvirus* genus. Almost all the *L. monocytogenes* phages listed in the INPHARED database belong to the *Pecentumvirus* genus, which indicates that these phages are most frequently isolated and sequenced- most likely correlating with their abundance in the environment and infection efficiency. *L. monocytogenes* is a Gram-positive bacterium with less diverse receptors than Gram-negative bacteria, which may explain why the phage products targeting *Listeria* contain very similar phages. Almost all phages infecting Gram-positive bacteria use cell wall carbohydrates, such as teichoic acids, as their binding receptors. *L. monocytogenes* phages bind to the N-acetyleglucosamine and rhamnose subunits of teichoic acids or to the peptidoglycan in the cell wall [139].

Although phages have enormous potential to be used as biocontrol agents in the environment and as therapeutics for eradicating pathogenic bacteria, there are several considerations that must be addressed before phages can be applied in these situations. The pharmokinetics and pharmodynamics of phages are far more complex than antibiotics and is typically phage dependent, which makes it difficult to generalise phages that are genetically similar [140]. For phages to successfully be used as therapeutics, further research into how they interact with the immune system in humans and animals must also be conducted. Additionally, administering phage can be problematic as they cannot survive the acidic pH of the stomach. One of the major issues with phage bioremediation is the emergence of bacterial resistance, however the likelihood that resistance emerges can be circumvented when using multiple phages in a phage cocktail [141].

## Conclusion

The majority of phages deployed for the biocontrol of pathogenic bacteria in the environment, and in humans and animals are dsDNA phages, due to their abundance in the environment. The lack of taxonomic diversity of phages utilised in One Health is attributed to isolation and selection bias, as phages are isolated for a specific host typically using a wastewater enrichment method. However, efforts to isolate novel phages, including ssDNA and RNA phages, must be improved to increase the diversity of phages utilised for these applications. This will, in turn, reduce the emergence of bacterial resistance and result in advancement of the One Health objectives leading to optimal health for all.

## Summary

- Currently used therapeutic/biocontrol phages represent only a minor fraction of global phage diversity.
- 98% (207 out of 211) of phages applied for One Health discussed in this review belong to the class *Caudoviricetes*, compared to 85% (24,298 out of 28,468) of sequenced isolated phages (INPHARED) belonging to this class.
- More diverse dsDNA phages should be isolated to reduce the emergence of bacterial resistance.
- The potential uses of ssDNA and RNA phages for advancing One Health objectives should be explored further.

## Supplementary Material

Supplementary Table S1-S3

## Data Availability

No primary data was generated in this review.

## Open Access

Open access for this article was enabled by the participation of John Innes Centre in an all-inclusive Read & Publish agreement with Portland Press and the Biochemical Society under a transformative agreement with JISC.

## Abbreviations

AMR: antimicrobial resistance
ARB: antibiotic resistant bacteria
ARG: antibiotic resistance gene
EMS: Early Mortality Syndrome
FDA: Food and Drug Administration
ICTV: International Committee on Taxonomy of Viruses
SARS: severe acute respiratory syndrome
STEC: Shiga-Toxin producing *Escherichia coli*
vOTUS: viral operational taxonomic units
WHO: World Health Organization
